# Film-reading workload in the West Midlands

**DOI:** 10.1186/bcr3287

**Published:** 2012-11-09

**Authors:** N Rogers, S Bradley, A Duncan, O Kearins, E O'Sullivan

**Affiliations:** 1 West Midlands Breast Screening Quality Assurance Reference Centre, Birmingham, UK; 2 South Birmingham Breast Screening Service, Birmingham, UK; 3 Warwickshire, Solihull & Coventry Breast Screening Service, Coventry, UK

## Introduction

The NHS Breast Screening Programme (NHSBSP) stipulates that film-readers should report a minimum of 5,000 cases per year. However, there is no stipulated maximum caseload. With the increasing number of women being included in the remit of the NHSBSP, the question of a maximum is becoming more relevant.

## Methods

Over the period 1 April 2008 to 31 March 2011 there were 37 radiologists and 34 advanced practitioners reporting breast screening cases within the West Midlands BSP. The range of screening cases reported during this period was between 35 and 49,053 per reader. Readers were divided into four groups according to total breast screening cases they had reported over the 3-year period (<15,000, 15,000 to 20,000, 20,000 to 25,000 and >25,000), in order to assess film-reader performance by number of cases reported. Only those who reported in the West Midlands for the full 3-year period were included (*n *= 47). This was to enable direct comparisons with Cornford and colleagues, who reported that performance in readers reporting >25,000 cases (over a 3-year period) may decline [1].

## Results

The group reading 20,000 to 25,000 cases had a significantly lower recall rate than the other three groups (*P *< 0.01) and a higher PPV (*P *< 0.01). The missed cancer rate and cancer detection rate showed no difference.

## Conclusion

The findings are not in agreement with previously published data suggesting that these parameters should not be applied as a quality standard to the NHSBSP. Performance will be continually monitored within the West Midlands over the coming years.
